# Alternative Strategies for Microbial Remediation of Pollutants via Synthetic Biology

**DOI:** 10.3389/fmicb.2020.00808

**Published:** 2020-05-19

**Authors:** Shweta Jaiswal, Pratyoosh Shukla

**Affiliations:** Enzyme Technology and Protein Bioinformatics Laboratory, Department of Microbiology, Maharshi Dayanand University, Rohtak, India

**Keywords:** synthetic biology, bioremediation, xenobiotics, genetic circuit, biosensor

## Abstract

Continuous contamination of the environment with xenobiotics and related recalcitrant compounds has emerged as a serious pollution threat. Bioremediation is the key to eliminating persistent contaminants from the environment. Traditional bioremediation processes show limitations, therefore it is necessary to discover new bioremediation technologies for better results. In this review we provide an outlook of alternative strategies for bioremediation via synthetic biology, including exploring the prerequisites for analysis of research data for developing synthetic biological models of microbial bioremediation. Moreover, cell coordination in synthetic microbial community, cell signaling, and quorum sensing as engineered for enhanced bioremediation strategies are described, along with promising gene editing tools for obtaining the host with target gene sequences responsible for the degradation of recalcitrant compounds. The synthetic genetic circuit and two-component regulatory system (TCRS)-based microbial biosensors for detection and bioremediation are also briefly explained. These developments are expected to increase the efficiency of bioremediation strategies for best results.

## Introduction

**FIGURE 1:**
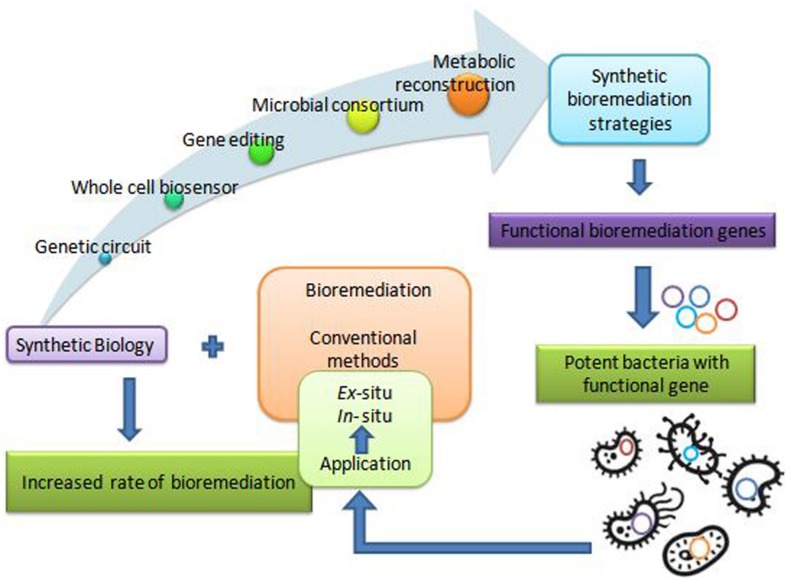
The strategies of synthetic biology applicable for bioremediation.

**FIGURE 2:**
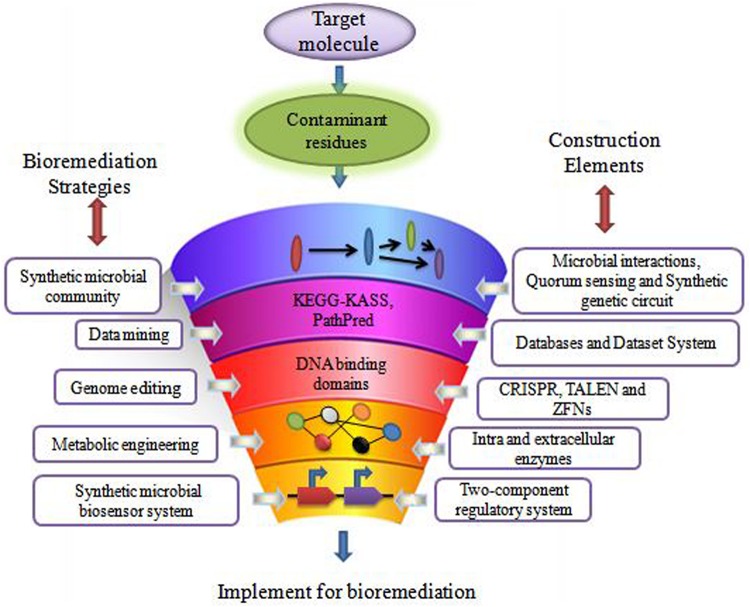
The components and their construction elements of synthetic biology for bioremediation studies.

The remediation processes aided by microorganisms present at the various contaminated scenarios constitute bioremediation (Basu et al., 2018; Kumar et al., 2019). Microbial remediation uses multiple metabolic pathways responsible for enzyme production (Sharma B. et al., 2018; Dangi et al., 2019). These enzymes mainly take part in the degradation pathways of xenobiotics (Junghare et al., 2019). There are different customary methods for bioremediation, primarily based on the site of bioremediation, *in* and *ex situ* (Tomei and Daugulis, 2013). *In situ* is applied to the site to minimize soil disturbance. This method is mostly adopted due to less expenditure from avoiding excavation and transport of contaminated soil (Khan et al., 2004). According to Khan et al., 2004 less disruption in *in situ* bioremediation causes less dust dispersion and hence better degradation (Joshi et al., 2016) of contaminant. Bioaugmentation, bioventing, biosparging, and engineered *in situ* bioremediation are main *in situ* bioremediation methods (Azubuike et al., 2016). *Ex situ* bioremediation methods are solid phase system (composting, landfarming, and biopiling) and slurry phase system (bioreactors) (Kumar et al., 2011). Transportation of soil to accelerate microbial degradation are done by solid and slurry phase systems, whereby treatments of domestic, industrial, and organic waste are done by *ex situ* bioremediation (Juwarkar et al., 2010). These traditional bioremediation methods take time and consume much cost expenditure, giving less result output. Traditional bioremediation (Duarte et al., 2017) processes showed the above limitations of extra time taking, less removal or dissimilation of pollutants, (Bharagava et al., 2019) disturbance to nature delicacy such as more land coverage for a long time, and a foul smell in the environment (Dangi et al., 2019; Kumar, 2019). Therefore, researchers are eager to discover new bioremediation technologies for best results. Dvoøák et al. (2017) described bioremediation via synthetic biology for boosting bioremediation strategies. This approach can catch the catabolic (Jacquiod et al., 2014) and metabolic complexities for reviewing the potential of the microbial population synthetically. The preliminary information for developing synthetic microbial models for bioremediation can be obtained by mining genes from the databases (Fajardo et al., 2019). The computer logics involvement can determine the microbial cell interactions with recalcitrant compounds (Kim et al., 2015). These strategies can together grasp the natural metabolic potential of microorganisms to transform into novel biological entities of interest (Dangi et al., 2019). Furthermore, the regulation of metabolic pathways (Alves et al., 2018) in a controlled manner can also be achieved for bioremediation processes (Rochfort, 2005). This transition via synthetic biology application (Figure 1) for remediation purposes would improve the bioremediation processes via the involvement of potent (Zhu et al., 2017) dissimilating particular contaminants (Trigo et al., 2008). Synthetic biological systems mediate cellular modulations for efficient functioning and working of existing processes. They permit the modification of cellular processes *viz.* metabolic pathway acting for a particular chemical compound. The advancement of synthetic biology for bioremediation of various contaminants is attaining the focus of scientists and researchers. For instance, a sustainable synthetic microbial community’s establishment for bioremediation is being investigated. Microbial interactions and quorum sensing within communities are vastly studied for application in the area of bioremediation with synthetic biology applications. Achievement of the synthetic genetic circuit of *Pseudomonas putida* proved to be the golden gadget for degradation studies. Besides this, genome editing by CRISPR-Cas, TALEN, and ZFNs adds knowledge for reviewing the progression in bioremediation studies. Synthetic microbial biosensors and metabolic engineering of cellular processes for utilization and detection of contaminant residues will remediate the environment from persistent recalcitrant pollutants. This review is focused on the above mentioned strategies and their elements (Figure 2) applicable for bioremediation purposes and research.

## Metabolic Reconstruction for Designing Synthetic Models

A computational platform is utilized for the reconstruction of cellular metabolism (Agapakis et al., 2012) via metabolic pathway analysis (MPA) (Banerjee et al., 2016). MPA mathematically represents the reactions of metabolism. This method is based on stoichiometric balance reactions so as to propose steady-state metabolic flux during cellular growth. The stoichiometry matrix imposes constraints of flux, making the consumption and production of the compound at a steady state (Bordbar et al., 2014). The maximum and minimum flux of a reaction can also be determined by providing the topper and least bound. This helps to define the extent of permissible flux supply (Richelle et al., 2016; Rawls et al., 2019). The next step is defining the objective according to the biological problem to be studied. This objective mathematically represents the reactions responsible for the phenotype appearance. The mathematical reactions and phenotype are combined with linear equations and solved by computational algorithms such as COBRA Toolbox11 and Matlab toolbox (Orth et al., 2010; Bordel, 2014). FBA is fundamentally simple, having immense applications in studying gaps, physiology, and genomes via systems biology approach (Kim et al., 2015; Hellweger et al., 2016). These gaps are missing metabolic reactions, making the genome partially known. FBA uses computational algorithms that can predict missing reactions *viz.* OptKnock and OptCom, which can knock out the genes responsible for producing the desired compound (Biggs et al., 2015). These approaches are beneficial for constructing microbial communities for bioremediation of particular contaminants (Khandelwal et al., 2013). However, MPA is the most challenging method when metabolic information is incomplete, making it difficult to obtain a real model (Covert et al., 2001). But this method shows cellular functions in the dynamic community, and thus is very useful for the prediction and exchange of metabolic flux in communities of microorganisms (Khandelwal et al., 2013). Recently, a metabolic model has been constructed by using two *Geobacter* species with parameterized electron transfer and metabolic exchange to characterize syntrophic growth dynamics. Such a system may have useful applications in the field of bioremediation and degradation of particular contaminants (Butler et al., 2010). A computational platform is also needed for better prediction of engineered genetic pathways for community dynamics. A graph-based tool Metabolic Tinker was developed by McClymont and Soyer to identify thermodynamically feasible biochemical routes for compounds deterioration (Johns et al., 2016). This may be applied to identify the routes for degradation of recalcitrant compounds by microbial consortia. These computational tools are utilized along with omics (Kim et al., 2014; El Amrani et al., 2015) and biological data for desired output (Berger et al., 2013) and toxicity prediction (i.e., META-CASETOX System) (Peijnenburg and Damborský, 2012). These are also applied for functional gene identification and their profile analysis, PCR analysis and drug discovery, etc (Dangi et al., 2019). Computer-aided drug discovery and development (CADDD) is used effectively with chemical and biological aspects, i.e., chemical structures accounting the biological role and its activity via ligand-based drug design, structure-based drug design, quantitative structure-property relationships, and quantitative structure-activity (Kapetanovic, 2008). Furthermore, Table 1 depicts similar methodologies applicable to bioremediation studies. De Jong (2002) analyzed the multicellular feedback control strategy in a bacterial consortium (Bruneel et al., 2011) to define the robustness conceivable

**TABLE 1:** The methodologies applied for bioremediation research.

**S.no.**	**Purpose**	**Approach**	**Methodology**	**References**
1.	Degradation study	Functional gene identification for bioremediation	PCR (Polymerase Chain Reaction) product sequence analysis	Jagwani et al., 2018
2.	Cell behavior study	Whole-cell simulation	A computer model for bacterial cell in response to the contaminated environment	O’Brien et al., 2015; Panigrahi et al., 2019
3.	Toxicity of chemicals	Analysis of chemical and biological properties	*In silico* toxicology (IST) protocols for toxicity assessment; QSAR (Quantitative Structure Activity Relationship) model	Pavan and Worth, 2006; Myatt et al., 2018
4.	Identification of functional bioremediating microbe	Target identification	Protein structure prediction, Protein – protein interaction (PPIs)	Shukla, 2017
5.	Remediation of textile dyes	Interaction of protein-ligand	Molecular docking	Sridhar and Chandra, 2014; Kumar et al., 2016
6.	Bioremediation of toxic pollutants	Structure prediction	Biodegradability evaluation and simulation system	Das et al., 2016

under desired conditions. They utilized an ordinary differential equations (ODE)-based model and agent-based simulation on a consortium (Hawley et al., 2014; Atashgahi et al., 2018) of interacting species population for increasing the efficacy of the proposed feedback control strategy. The application of bioinformatics (Arora and Bae, 2014) resources is a prerequisite dimension for obtaining the data to begin the microbial bioremediation studies of recalcitrant compounds (Gong et al., 2012; Ofaim et al., 2019). This involves the information related to the degradation of xenobiotics by microbes and their pathways for dissimilation (Dao et al., 2019; Salam and Ishaq, 2019; Thelusmond et al., 2019; Wei et al., 2019). The data related to end products and intermediate metabolites released throughout degradation pathways can also be retrieved (Dvoøák et al., 2017). An extended information source linked to degradation is MetaRouter, allowing data (Singh and Gothalwal, 2018) for life sciences laboratories to explore degradation possibilities of recalcitrant compounds (Mohanta et al., 2015). The information on oxygenic degradation of xenobiotics can be retrieved from OxDBase, a biodegradative oxygenase database (Chakraborty et al., 2014; Shah et al., 2018). Oxygenase is a class of enzyme which transfers the oxygen molecule for oxidizing the chemical compound. They play a role in the degradation of organic compounds by aromatic ring cleavage (Jadeja et al., 2014). OxDBase is very particular in providing knowledge of oxygenases-catalyzed reactions, and is a powerful tool applicable to bioremediation studies (Singh, 2018). Bioconversion and biodegradation of persistent and toxic xenobiotics (Desai et al., 2010; Bao et al., 2017) compounds catalyzed by oxygenases decrease the compound sustainability and toxicity in the environment (Kües, 2015; Kondo, 2017). Therefore, OxDBase is very helpful in acknowledging the degradation processes involved in bioremediation (Shah et al., 2012). The transcriptional characterization of genes responsible for the biodegradation and biodissimilation of a particular compound has great significance in proposing molecular methodologies. This can be done by the Bionemo (Biodegradation Network Molecular Biology) database (Libis et al., 2016a). Bionemo contains the entries for sequences of genes coding for biodegradation (Carbajosa and Cases, 2010). It also links the gene transcription and its regulation (Libis et al., 2016b). The data retrieved from Bionemo can be used for designing cloning experiments and primers (Arora and Shi, 2010). Garg et al. (2014) used eMolecules and the EAWAG-BBD PPS database for the prediction of pathways involved in the biodegradation of 1-naphthyl-N-methyl carbamate. These above findings empower the researchers to analyze and establish what prerequisites must be fulfilled for developing synthetic bioremediation models.

## Designing the Synthetic Microbial Communities

Recent advancements in the field of synthetic biology for environmental issues have shown a great impact. The use of GMOs in environmental biotechnology for remediation (Malla et al., 2018) of toxic compounds, xenobiotics, and pesticidal compounds are being done. To design a synthetic community, it is important to understand natural microbial communities (Schloss and Handelsman, 2008). In a natural community, it is difficult to find out which species are actually taking part in bioremediation (Großkopf and Soyer, 2014). Thus, a synthetic microbial community is a promising method for constructing an artificial microbial community with function-specific species for bioremediation purposes. These communities may act as a model system for the study of functional, ecological, and structural characteristics in a controlled manner. Großkopf and Soyer (2014), defined synthetic community by the culturing of two microbial species under well-defined conditions on the basis of interaction and function (Bruggeman and Westerhoff, 2007). These factors determine the dynamics and structure of the community. It is based upon the identification of processes and patterns engaged in by bacterial species. These microbial interaction patterns are metabolism-driven and responsible for community interaction (Wintermute and Silver, 2010). Social-based microbial interactions (i.e., mutualism, cooperation, and competition, etc.) and the total outcome of these interactions between two microbial populations can be +/+ and −/+ or +/−, respectively (Foster and Bell, 2012). It is said that community structure and function majorly depends on cooperation. The effect of cooperation on community dynamics is determined by engineered cooperation resulting in the synthetic community. Engineered cooperation between two microbial strains (Singh et al., 2016) can be done by manipulation of environmental conditions, i.e., knocking the genes out and in Zuroff et al. (2013). Beyond this, other interaction patterns have been analyzed with engineered microbial species in the synthetic community. Such an application of engineered interaction is highly recognizable in bioremediation strategies (Sharma, 2012). Synthetic biology provides greater potential for the sustainable existence of microorganisms (Dellagnezze et al., 2014) acting together in a large population. Thus, synthetic microbial communities are proved as a key strategy for the bioremediation of contaminants, i.e., pesticides, petroleum (Kachienga et al., 2018), oil spill, acid drainage (Serrano and Leiva, 2017), etc. For building the synthetic microbial communities, the engineered interspecies and intraspecies interactions can make cellular functions robust and enhance the capabilities of microbial consortia in various contaminated scenarios. Quorum sensing is a bacterial signaling mechanism, which is a density-dependent phenomenon via cell-cell communication and population level behavior. The signaling is done by the release and reception of chemical compounds by microbial candidates in a population. This leads to multicellular behavior (Obst, 2007), offering engineerable tasks to design function specific synthetic communities. These synthetic models can also be exploited to obtain a rational design that can lose the function when subjected to competition with other species in the natural environment. With the evolution of genomic constituents and gene transfer, the possibility of the gradual extinction of genetic circuits is present. Thus, strategies are required to maintain the robustness of the synthetic community, achieved via the synthetic models by the development of synergistic and cooperative properties that reduce instability and loss of function (Johns et al., 2016). A recent study by Coyte et al. (2015) suggests that competition among species is significant in determining the stability of communities, acting as a limiting factor in the stability of the synthetic community. Thus, these dynamics must be accelerated in order to design particular function specific synthetic communities for bioremediation purposes (Coyte et al., 2015).

## Genetic and Metabolic Engineering

**FIGURE 3:**
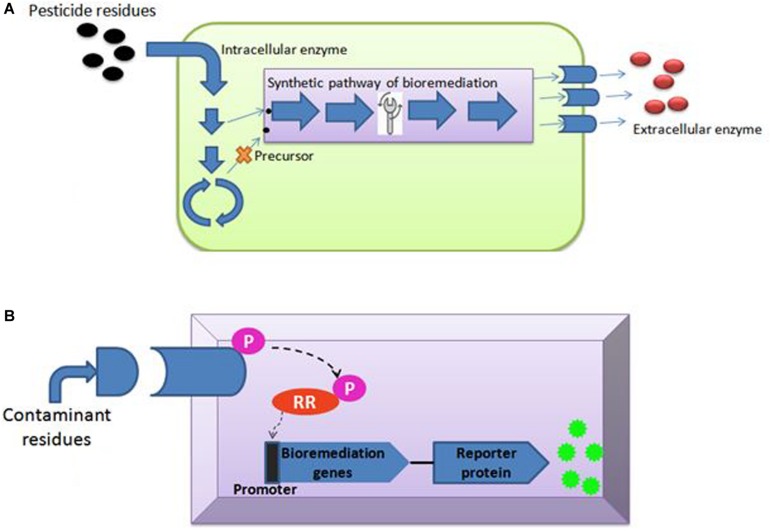
Schematic presentation **(A)** intracellular and extracellular enzymes production; **(B)** TCRS based biosensor.

Enríquez (2016) said that genome editing is an umbrella term that refers to “scientific technological advances that enable rational genetic engineering at a local (gene) or global (genome) level to facilitate precise insertion, removal, or substitution of fragments of Deoxyribonucleic acid (“DNA”) molecules, comprising one or more nucleotides into the cell(s) of an organism’s genome.” Transcription-activators like effector nucleases (TALEN), clustered regularly interspaced short palindromic repeats (CRISPR-Cas), and zinc finger nucleases (ZFNs) are major gene editing tools used (Table 2). The most efficient and simple technique of gene editing has been described as CRISPR-Cas (Kanchiswamy et al., 2016). These tools can boost the process of bioremediation. TALEN has a DNA-binding modular which is sequence-specific for the host genome (Utturkar et al., 2013). TALEN binding to DNA creates a double stranded break (DSB) in the target sequence and leaves sticky ends for stability. Similarly, ZFNs is also a DNA-binding domain composed of 30 amino acids. It introduces DSB at the target site of the host genome by the Fok1 cleavage domain. A new sense of using hybrid nucleases containing TALENs and ZFNs nucleases came to act for solving the molecular complications. The CRISPR-Cas system, on the other hand, has unique action properties of high sequence specificity and multiplex gene editing. This unique property is derived from bacteria *Streptococcus pyogenes* as immunity against the virus. The CRISPR-Cas system consists of crisper derived RNA (crRNA) and trans acting antisense RNA (trcRNA) joined by guide RNA (gRNA). gRNA directs the Cas9 enzyme to introduce DSB in the target DNA sequence by recognizing it. These gene editing tools create the knock-in and knock-out and are under processing for implementation in bioremediation studies (Kumar V. et al., 2018). Recent reports indicate though that the CRISPR-Cas system is mostly adopted and performed by researchers in model organisms i.e., *Pseudomonas* (Karimi et al., 2015; Nogales et al., 2020) or *Escherichia coli* (Chen et al., 2018; Marshall et al., 2018; Pontrelli et al., 2018). Nowadays, the new insights toward CRISPR tool kits and designing gRNA for expression of function-specific genes related to remediation in non-model organisms (i.e., *Rhodococcus rube*r TH, *Comamonas testosteroni* and *Achromobacter* sp. HZ01) are also suggested in the field of bioremediation (Mougiakos et al., 2016; Jusiak et al., 2016; Hong et al., 2017; Stein et al., 2018; Tang et al., 2018; Liang et al., 2020). For gene editing and metabolic engineering, the contaminant-inhabited bacteria are the most suitable candidates because they are used to survive and harbor in stress conditions of various toxic, recalcitrant and non-degradable xenobiotics, as discussed above. Moreover, understanding metabolic pathways seems to be important in studying the microbial bioremediation (Plewniak et al., 2018), i.e., bioremediation of toxic pollutants by the haloalkane dehalogenases production pathway and decontamination of pyrethroid from the soil via the biodegradation pathway of fenpropathrin studied in *Bacillus* sp. DG-02 (Chen et al., 2014). Metabolic engineering leads to modification of the existing pathway for the betterment of the bioremediation process (Michel et al., 2007). This approach majorly covers the study of microbial enzymes, i.e., oxidases, esterases, monooxygenases, oxidoreductases, phenoloxidases involved at various degradation steps (Figure 3A; Mónica and Jaime, 2019; Mujawar et al., 2019). Enzyme-based bioremediation has many advantages because it is an eco-friendly process. The genetic approach increases the perspective of getting recombinant enzymes. There are research reports of extracellular enzymes having a role in enzymatic bioremediation. For instance, arsenic bioremediation (Andres and Bertin, 2016; Choe and Sheppard, 2016; Akhter et al., 2017; Biswas et al., 2019) (bioaccumulation and biotransformation) is achieved *via* arsenite oxidase coded by *aioA* gene of *Klebsiella pneumonia* (Mujawar et al., 2019); enzymes released by white rot fungi degrade PAHs (polycyclic aromatic hydrocarbon) (Zhao and Poh, 2008; Košnár et al., 2019), dyes, TNT (2,4,6- Trinitrotoluene) and PCBs (polychlorinated biphenyls) (Gupte et al., 2016; Kutateladze et al., 2018; Sadańoski et al., 2018). Esterase D enzyme acts on β-endosulfan (organochlorine pesticide), producing simpler compounds (Mehr et al., 2017; Chandra et al., 2019). *LiPs* encoding hemoproteins from *Phanerochaete chrysosporium* degrade PAHs. However, incomplete or partial degradation of contaminants lead to simpler non-toxic degradable compounds which can be consumed by microbes (Kumavath and Satyanarayana, 2014) as intermediates in biological pathways or substrate, i.e., *LiP* (lignin peroxidase) dissimilate benzopyrene to three compounds of quinine, namely 1,6- quinone, 6,12- quinine and 3,6- quinine (Gupta and Pathak, 2020). Furthermore, *MnP* (Manganese peroxidase) oxidizes organic compounds in the presence of Mn(II) (Xu et al., 2018; Singh et al., 2019). Laccase, MFO (mixed function oxidases), glutathione S transferase, cytochrome P_450_ also acts in biodegradation of recalcitrant compounds (Singh, 2019; Boudh et al., 2019). Catechol 1,2-dioxygenase (intracellular enzyme) from *Pseudomonas* NP-6 dissimilate catechol to muconate compounds (Guzik et al., 2011). Also, enzyme immobilization (Cavalca et al., 2013; Sharma B. et al., 2018) increases the half-life, stability, and enzyme activity at a notable level. The enzymatic bioremediation is an elementary, expeditious, and environmental friendly method for microbial removal and degradation of persistent xenobiotics compounds (Sharma B. et al., 2018). Isolation and characterization of microorganisms with enzymatic capabilities have been done with the limitation of less productivity (Rayu et al., 2012). Organophosphates (OP) and organochlorines (OC), major constituents of insecticides, accumulate in the agricultural soil (Panelli et al., 2017) and reach the water bodies via agricultural run-off. Effective bioremediation of γ-hexachlorocyclohexane (OC) and methyl parathion (OP) has been reported by genetically engineered microorganisms (Gong et al., 2016). Moreover, bioremediation of organophosphates and pyrethroids has been experimented with using genetically modified *P. putida* KT2440 (Zuo et al., 2015). With the advent of metabolic engineering, the catabolism and degradation of various persistent compounds has been reported. The degradation pathways of methyl parathion and γ-hexachlorocyclohexane in *Sphingobium japonicum* and *Pseudomonas* sp. WBC-3 witnessed the bioremediation strategy (Liu et al., 2005; Miyazaki et al., 2006). Furthermore, 1, 2, 3-trichloropropane, a persistent constituent of fumigant, is dissimilated into the environment (Techtmann and Hazen, 2016) via heterologous catabolism by the assembly of three enzymes from two different microorganisms in *E. coli* (Dvorak et al., 2014). A metabolic pathway (Bertin et al., 2011) degrading organophosphorus and paraoxon is engineered by inserting the organophosphorus hydrolase gene (opd) and pnp operon encoding enzymes that convert p-nitrophenol into β-ketoadipate in *P. putida* (de la Pena Mattozzi et al., 2006). A study showed *pobA* and *chcpca* gene clusters of *Rhodococcus opacus* R7 take part in the bioremediation of naphthenic acid; more specifically, expression *aliA1* gene codes for fatty acid CoA ligase for degrading long chains of linear as well as alicyclic naphthenic acid (Zampolli et al., 2020). To minimize the accumulation, the above-mentioned strategy is attained using microbes for partial or complete mineralization of persistent compounds (Miyazaki et al., 2006).

**TABLE 2:** Comparative features of CRISPR, TALE, and ZFNs.

**Features**	**Gene editing tools**			**References**
	**CRISPR**	**TALEN**	**ZFNs**	
System	Adaptive immune system	Pathogenic *Xanthomonas*	Gene expression system	Kumar N. M. et al., 2018
Specificity	crRNA	TALE Domain	Zn finger Domain	Jaiswal et al., 2019b
Cleavage	Cas9	*Fok*I nuclease	Nuclease	Kumar V. et al., 2018; Jaiswal et al., 2019b
Nucleases per target per experiment	Single or more sgRNA; singleCas9	Single TALEN pair	Single ZFN pair	Hamilton et al., 2019
Activity	High	High	Moderate	Shanmugam et al., 2019
Designing and screening	Easy	Difficult	Difficult	Dangi et al., 2019
Multiple gene editing	Suitable	Not suitable	Not suitable	Sinha et al., 2019

## Synthetic Genetic Circuit and Microbial Biosensor

The synthetic genetic circuit requires chassis for implantation. The *P. putida* is a HVB (Host Vector Biosafety) strain recognized as safe by the Recombinant DNA Advisory Committee. It is also referred to as GRAS (Generally Recognized as Safe) to release in the environment. It is ideal for the next generation of synthetic biology chassis panel because it can withstand high intolerant changing conditions including temperature, pH, solvents, toxins, osmotic, and oxidative stress. Also, *P. putida* has versatile metabolism and low nutrient requirements (Pabo and Nekludova, 2000). These qualities make this organism the best bacterial model for environmental bioremediation applications (Tanveer et al., 2018). Recently, the *P. putida* synthetic genetic circuit has been established for the designing of promoter genes and expression of the gene responsible for the degradation of persistent compounds (Adams, 2016). An extension of synthetic biology is the integration of genome with reporter system, and synthetic promoters of *P. putida* may be developed for synthetic bioremediation pathways. Elmore et al. use serine integrases for synthetic genetic circuit development. Microbial cells have the advantage of a cellular system, which controls cell growth and response to external factors like temperature, light, pH, and oxygen (Tropel and Van Der Meer, 2004). The external environment of microbes inhabiting the contaminated site will respond to concentrations of various persistent compounds present (Ray et al., 2018; Antonacci and Scognamiglio, 2019). Whole cell biosensors for checking the presence, detection and biodegradation potential of xenobiotics compounds (pharmaceutical residues, pesticides, paraffin, PAHs and PCBs, etc.) present (Adhikari, 2019) in environmental samples are attaining attention (Wynn et al., 2017; Heng et al., 2018; Patel et al., 2019). The reporter proteins acting microbe makes a color signal at the detection of particular contaminants *via* transducer (Zhang and Liu, 2016). A biosensor aiming for detection and bioremediation purposes must have enhanced contact between microbe and contaminant (Dhar et al., 2019). This helps the bacterium to adjust their cellular pathways in response to external environmental conditions and encodes the genes for utilizing the recalcitrant compounds as substrate (Bilal and Iqbal, 2019; Skinder et al., 2020). Synthetic biology strategies are feasible for removing a particular toxic compound because the genetic circuits can be developed against the exogenous environmental toxin (Checa et al., 2012; Tay et al., 2017). The synthetic genetic circuits are assembled via a two-component regulatory system (TCRS) in bacteria (Futagami et al., 2014; Uluşeker et al., 2017). This system acts upon environmental change, and thereby, cells respond to these changes. A prokaryotic TCRS has histidine kinase (HK) and response regulator (RR). The HK is a sensor domain with an extracellular loop present as an integral membrane protein. HK also has a transmitter domain in the last cytoplasmic transmembrane, which is a highly conserved domain. Histidine phosphotransfer (DHp) and catalytic ATP-binding domain (CA) acts for molecular recognition of RR and ATP hydrolysis. The transmitter domain transmits the signal from periplasm to cytoplasm via PAS (Periodic circadian proteins, Aryl hydrocarbon nuclear translocator proteins, and single minded proteins), HAMP (HKs, Adenyltatecyclases, Methyltransferases, and Phosphodiesterases) and GAF (cGMP-specific phosphodiesterases, adenylyl cyclases, and formate hydrogenases) (Casino et al., 2010). Thus, HK senses the external environmental change and adds phosphate to conserved histidine. The HK also regulates RR by phosphorylating the aspartate residues. This promotes the promoter (Figure 3B) binding to activate the gene expression or vice-versa (Ravikumar et al., 2017). Therefore, TCRS-based synthetic biology application for biosensor development for cell-mediated detection and bioremediation can prove to be a new advancement.

## Ecological Safety and Risk Assessment

The scientists and researchers are performing the experimental setup to study the bioremediation (Yergeau et al., 2012) potential against various pollutants like oil spill, plastics, synthetic dyes, organic hydrocarbons (Yadav et al., 2015), pesticides (Jaiswal et al., 2019a), heavy metals (Hemmat-Jou et al., 2018; Lebrazi and Fikri-Benbrahim, 2018), and other xenobiotics, etc (Rucká et al., 2017; Paniagua-Michel and Fathepure, 2018; Wu et al., 2020). Considering that bioremediation is performed in an open environment rather than in a closed fermentation tank, the ecological safety of bioremediation performing bacteria must be considered. Economic safety is justified by the metabolic aptness (Gillan et al., 2015) of microorganisms as compared to other traditional physical and chemical bioremediation methods. Besides, regulation for using genetically and metabolically modified bacteria is released to evaluate the possible risks (Khudur et al., 2019). The risk assessment is mainly done by regulatory agencies, i.e., Organization for Economic Cooperation and Development (OECD) at the application level for environmental safety (Russo et al., 2019; Alam and Murad, 2020; Pastor-Jáuregui et al., 2020). The possible risks are gene contamination in the native member of microbial consortium, leading to mislaying of the natural trait (Mills et al., 2019; Pineda et al., 2019; Rycroft et al., 2019). The competitiveness between natural and genetically modified species can give rise to selection pressure on non-target microflora (Kumar N. M. et al., 2018; Mohapatra et al., 2019). Moreover, environment and ecosystem risk assessments infer unpredictable and adverse effects, as discussed above (Cervelli et al., 2016; van Dorst et al., 2020). Particularly, the ecological risk assessment behind addition of GEMs (Genetically Engineered Microorganisms) (Benjamin et al., 2019; Ahankoub et al., 2020) to the native environment rather than a laboratory (Fernandez et al., 2019) is done mainly because of unnecessary delivery of antibiotic resistance marker along with recombinant genome of interest (Davison, 2002; Nora et al., 2019), and unintentional uptake or transfer of induced genes to other microorganisms (French et al., 2019; Janssen and Stucki, 2020). This phenomenon is definately disturbing the microbial native genome entity (Gangar et al., 2019; Petsas and Vagi, 2019). Another aspect come into considerance, the change in microbial metabolism (Okino-Delgado et al., 2019), will release uncertain toxic compounds for the environment and health, indirectly acting as opposition microbial candidates in this context (Myhr and Traavik, 2002). Under the TSCA (Toxic Substances Control Act) (Gardner and Gunsch, 2020), the Office of Pollution Prevention and Toxics (OPPT) programs (Pietro-Souza et al., 2020) of the United States Environmental Protection Agency (Leong and Chang, 2020; Saxena et al., 2020) moniters the environmental and health risks and releases premanufacture legal notice for the accreditation of field research outlines and prototypes (McPartland and McKernan, 2017; Khan et al., 2020). A magnificant example is given by University of Tennessee. In 1995, they gave application and suggested the risk evaluation of microbial bioremediation agents (mainly *Pseudomonas fluorescens* HK44) on the environment and health (Sayler et al., 1999; Khan et al., 2016; Sharma J. K. et al., 2018; El Zanfaly, 2019). Most remarkable is that the literature survey points toward a biowar weapon for humanity (Gómez-Tatay and Hernández-Andreu, 2019; Wang and Zhang, 2019), stating that gene editing tools left in bad hands could mislead ethical and moral duties (Khan, 2019; Thakur et al., 2019; Sharma et al., 2020).

## Conclusion and Future Perspectives

The microbial bioremediation process for removal and detoxification of contaminants from the environment has now emerged as the best option. Synthetic biology is addressing the decontamination and remediation strategies for xenobiotics and related compounds from the environment. It has been observed that the requisite for understanding existing metabolic pathways is a must for developing synthetic models of bioremediation. Moreover, genomics reconstruction methods (Luo et al., 2014; Marco and Abram, 2019) and technologies made a solid platform for bioremediation studies. Satisfactory progress has been witnessed in the field of bioremediation among various contaminants with the role of specific genes and enzymes applicable via synthetic biology methodologies. Therefore, it is concluded that microbial synthetic biology remediation strategies not only increase the efficiency of microbial bioremediation processes for a particular contaminant, but also provide the best methodologies for researchers.

## Author Contributions

SJ contributed to this article under the guidance of PS. SJ acknowledges the support and guidance of PS in pursuing a doctorate under his guidance.

## Conflict of Interest

The authors declare that the research was conducted in the absence of any commercial or financial relationships that could be construed as a potential conflict of interest.
